# Role of thyroid hormone-integrin αvβ3-signal and therapeutic strategies in colorectal cancers

**DOI:** 10.1186/s12929-021-00719-5

**Published:** 2021-04-08

**Authors:** Yu-Chen S. H. Yang, Po-Jui Ko, Yi-Shin Pan, Hung-Yun Lin, Jacqueline Whang-Peng, Paul J. Davis, Kuan Wang

**Affiliations:** 1 Joint Biobank, Office of Human Research, Taipei Medical University, Taipei, 11031 Taiwan; 2 School of Medicine, I-Shou University, Kaohsiung, 84001 Taiwan; 3 Department of Pediatrics, E-DA Hospital, Kaohsiung, 82445 Taiwan; 4 Graduate Institute of Nanomedicine and Medical Engineering, College of Medical Engineering, Taipei Medical University, Taipei, 11031 Taiwan; 5 Graduate Institute for Cancer Biology and Drug Discovery, College of Medical Science and Technology, Taipei Medical University, Taipei, 11031 Taiwan; 6 Cancer Center, Wan Fang Hospital, Taipei Medical University, Taipei, 11031 Taiwan; 7 Traditional Herbal Medicine Research Center of Taipei Medical University Hospital, Taipei Medical University, Taipei, 11031 Taiwan; 8 TMU Research Center of Cancer Translational Medicine, Taipei Medical University, Taipei, 11031 Taiwan; 9 Pharmaceutical Research Institute, Albany College of Pharmacy and Health Sciences, Albany, NY 12144 USA; 10 Albany Medical College, Albany, NY 12144 USA

**Keywords:** NDAT, Colorectal Cancer, Integrin αvβ3, Epidermal growth factor receptor, Ras mutation

## Abstract

Thyroid hormone analogues—particularly, l-thyroxine (T_4_) has been shown to be relevant to the functions of a variety of cancers. Integrin αvβ3 is a plasma membrane structural protein linked to signal transduction pathways that are critical to cancer cell proliferation and metastasis. Thyroid hormones, T_4_ and to a less extend T_3_ bind cell surface integrin αvβ3, to stimulate the extracellular signal-regulated kinase 1/2 (ERK1/2) pathway to stimulate cancer cell growth. Thyroid hormone analogues also engage in crosstalk with the epidermal growth factor receptor (EGFR)-Ras pathway. EGFR signal generation and, downstream, transduction of Ras/Raf pathway signals contribute importantly to tumor cell progression. Mutated *Ras* oncogenes contribute to chemoresistance in colorectal carcinoma (CRC); chemoresistance may depend in part on the activity of ERK1/2 pathway. In this review, we evaluate the contribution of thyroxine interacting with integrin αvβ3 and crosstalking with EGFR/Ras signaling pathway non-genomically in CRC proliferation. Tetraiodothyroacetic acid (tetrac), the deaminated analogue of T_4_, and its nano-derivative, NDAT, have anticancer functions, with effectiveness against CRC and other tumors. In *Ras*-mutant CRC cells, tetrac derivatives may overcome chemoresistance to other drugs via actions initiated at integrin αvβ3 and involving, downstream, the EGFR-Ras signaling pathways.

## Introduction

Colorectal cancer (CRC) is the second leading cause of cancer death worldwide and studies of CRC understandably attract much attention in the oncology literature [108]. New therapeutic targets in the tumors and expanded anticancer drug choices have importantly transformed treatment strategies for CRC in recent years. Improved patient outcomes have resulted over the past two decades [12, 113]. Improvements in surgical techniques for managing the oligometastatic disease of lungs and liver in CRC have also contributed to improved overall survival (OS) of CRC patients. 5-Fluorouracil (5-FU) has increased CRC OS from 14.2 to nearly 30 months when combined with folinic acid, fluorouracil, oxaliplatin (FOLFOX)- and folinic acid, and irinotecan (FOLFIRI)-based chemotherapies [53]. However, this improvement has not increased the 5-year survival rate for patients with Stage IV disease; the rate remains at < 15%, and metastatic CRC (mCRC) remains essentially incurable [103].

Among the new therapeutic targets in mCRC that appear to have promising effects are *Ras* isoforms. *Ras* genes are the most frequently mutated family of oncogenes in cancer. CRCs often contain mutant Ras proteins and these appear to be linked to chemoresistance. However, most Ras-specific targeted therapeutic strategies have to-date been unsuccessful [11]. No K-Ras-specific drugs have been approved for clinical use, although AMG510 is a therapeutic option for patients with KRAS G12C mutations [101]. New therapeutic approaches are needed for Ras-mutant CRC. Studies from our group and others indicate that cell surface integrin αvβ3 may play important role in regulation of CRC proliferation, especially under influence of thyroid hormones [26, 64, 69, 88]. Signaling induced by thyroid hormone via integrin αvβ3 may be involve crosstalk with epidermal growth factor receptor (EGFR)-Ras and contribute to the development of CRCs.

### Integrin αvβ3 signal and genomic actions of thyroid hormone in CRC

Traditional genomic actions of thyroid hormone start with intranuclear binding of the hormone by nuclear thyroid hormone receptors (TRs) that are transcription factors [8]**.** In the genomic actions of thyroid hormone, T_4_ serves as a prohormone for T_3_ and the latter is the principal ligand of TR proteins. Triiodothyronine has a tenfold higher affinity than that of T_4_ for nuclear receptors [100]**.** The complex of TRβ with T_3_ translocates to the nuclear compartment where it sheds associated co-repressors, attracts co-activator proteins and becomes transcriptionally active. Although T_4_ involve in the initiation of this process of co-repressor releasing, it does not start the transcription[25]. Evidence indicates that traditional TRβ1-T_3_ plays negative role in cancer cell proliferation (Table 1) [64]. Table 1 lists a number of these overlapping genomic and nongenomic functions of thyroid hormone. On the other hand, the extracellular T_4_ or to a less extend T_3_ can, via a specific receptor on a plasma membrane integrin αvβ3, activate extracellular signal-regulated kinses (ERK1/2) and downstream signal transduction pathways to promote cell proliferation in variety types of cancer cells [6, 13, 33, 58, 71, 84].

**Table 1:** Overlapped Genomic and Non-Genomic Actions of Thyroid Hormones

	Genomic actions	Non-genomic actions
Integrin αvβ3	No	Yes [22]
ERK1/2 activation	No	Yes [105]
PI3K activation	No	Yes, only T_3_ activates PI3K [17]
T_4_-induced integrin αvβ3 internalization	No	Yes, nuclear phosphorylated αv monomer-MAPK-p300 complex binds to the promoter region of a panel of genes [70]
Nuclear receptor TRβ1 involvement	Yes	No
Shuttling ERα and TR to nucleus	No	Yes [75]
Actin-reorganization	Yes, thyroid hormone regulates actin expression [14]	Yes [20]
Gene expression	Dependent	Dependent but without ligand-TR complex [64, 70]
Regulating TRβ1 expression	Yes	Yes, T_4_ via αvβ3 regulates post-translational modifications of TRβ1[67]
Thyroid hormone-induced cancer cell proliferation	Yes, TRβ1 expression inhibits cancer proliferation [98]	Yes, integrin αvβ3-dependent [74]

The integrin αvβ3 is one of two dozen integrin heterodimers found on the surfaces of cells. While it has an important role in maintaining normal cell structure and in signal transduction, the integrin αvβ3 was shown to be over-expressed in high-growth endothelial cells and solid tumor and leukemic cells [9, 10, 23, 24, 26, 37, 42, 43, 64, 90]. Several small molecules (resveratrol[10], non-peptide hormones like steroid hormones [10] and thyroid hormones (T_4_, T_3_) have specific binding sites (receptors) on integrin αvβ3; at these sites, the ligands induce signal transduction and sequentially stimulate biological activities on cancer and endothelial cells [10]. These activities include cell proliferation [12, 20].

At physiological concentrations, thyroid hormone (T_4_) but not T_3_ [12, 20] initiates at the iodothyronine receptor on cell surface integrin αvβ3. As noted above, T_4_ via the integrin activates downstream ERK1/2, but the hormone, itself, does not enter the cell as a part of these functions. The consequences of signals generated at the integrin by T_4_ in cancer cells include cell proliferation, anti-apoptosis and radioresistance [12, 20], as discussed in the sections below. After interacting with T_4_, integrin αvβ3 is endocytosed into cytoplasm. Integrin monomeric αv, but not β3, translocates to the nucleus [70] and may function as a co-activator protein.

The interaction between thyroid hormone and integrin αvβ3 has been revealed by Davis’ group using computational modeling [65]. An arginine-glycine-aspartate (RGD) recognition site on the heterodimeric integrin αvβ3 is essential to the binding of a variety of extracellular matrix proteins. RGD peptides block the thyroid hormone binding site on integrin αvβ3 to inhibit and consequent ERK1/2 activation. These observations suggest that the hormone interaction site is located at or near the RGD recognition site on integrin αvβ3. Focal adhesion kinase (FAK) is a non-receptor tyrosine kinase that promotes cell migration and invasion through the control of focal adhesion turnover. Downstream of integrin αvβ3, FAK connects ERK1/2[104], PI3K/AKT[77], and other signal transduction pathways.

### Thyroid hormone binds to integrin αvβ3 to promote cancer proliferation

**Fig. 1:**
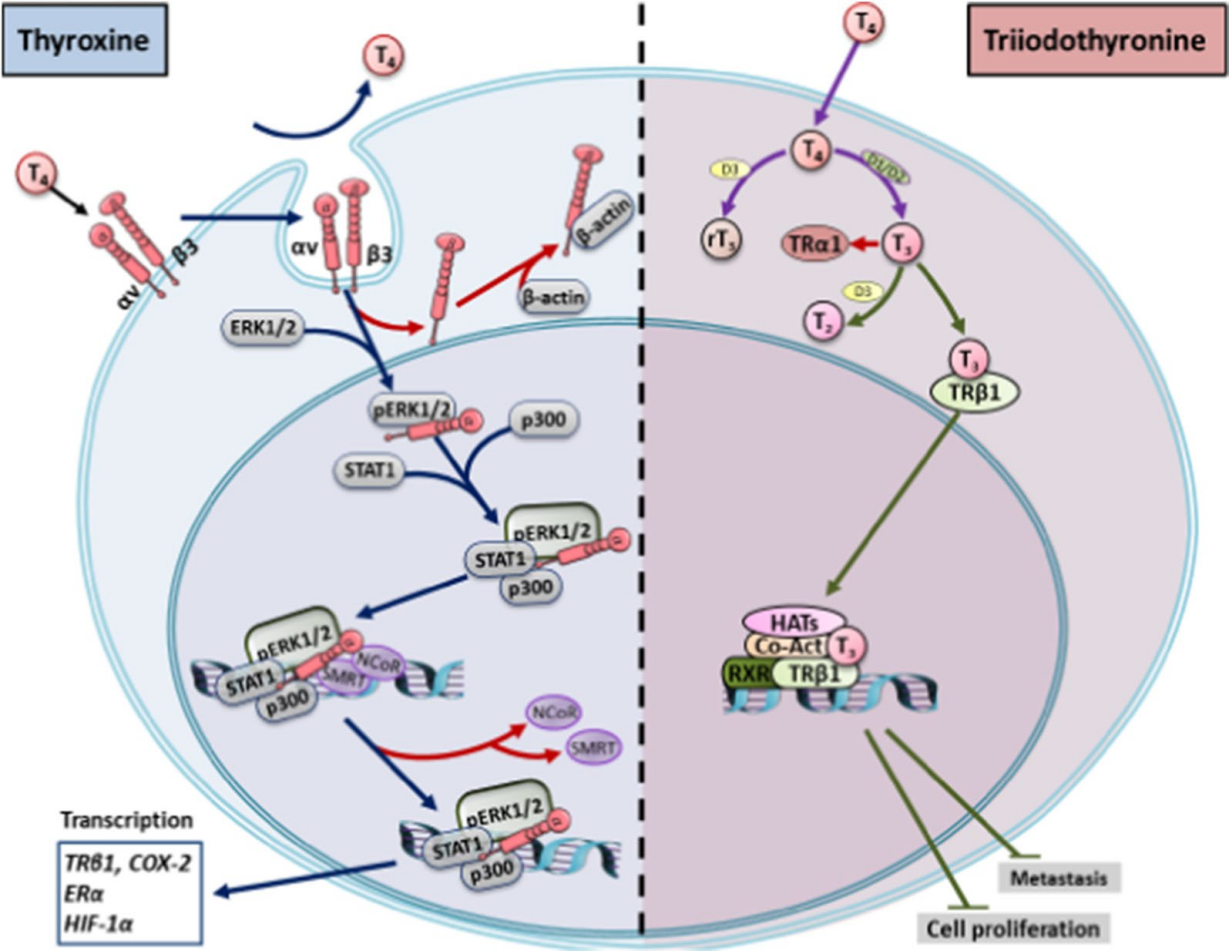
Thyroxine and Triiodothronine induce gene expression via different pathways. Thyroid hormone (T_4_) binds with integrin αvβ3 to induce integrin αvβ3 endocytosis without T_4_ bound. The integrin αvβ3 in cytoplasma associates with activated ERK1/2. The integrin αv monmer-pERK1/2 translocates into the nucleus and forms transcriptional complex with p300 and pSTAT3 which releases co-repressors, NCoR and SMRT from promotor region. The integrin αv-pERK-STAT3-p300 complex plays a co-activator function. On the other hand, T_4_ can also penetrate cell membrane via active transporters, and converted to T_3_ by deiodinase (D1 or D2). T_3_ binds to TRβ1, and the consequences are normal thyroid hormone-dependent biological activities which also show anti-proliferative effect in cancer cells

At physiological concentration, T_4_, but not T_3_, interacts with integrin αvβ3 to induce integrin αvβ3 to translocate into cytosol without T_4_ companion [70]. Several studies indicate that there are multi-mechanisms regulating integrin internalization [27]. Integrin αvβ3 has been shown to be internalized through caveolin-dependent mechanisms [35]. A possible mechanism is that endocytosed integrin αvβ3 is phosphorylated and binds with caveolin during endocytosis [117]. Sequentially, integrin β3 disassociates from complex, and the integrin α/caveolin complex binds with phosphorylated ERK1/2 [55]. The activated integrin αv-ERK1/2 complex translocates into nucleus and regulates transcriptional activities via binding to other transcription factors [120]. T_4_ induces nuclear integrin αv-ERK1/2-complex further associates with transcriptional coactivators, p300 and STAT1, and with corepressors, NCoR and SMRT[70]. The complexed phosphorylated ERK1/2 may be response to phosphorylation of coactivators [82] and corepressors [25]. Phosphorylation activates functional co-activators and repressors. The complex binds promotors of responsible genes including estrogen receptor-α, cyclooxygenase-2, hypoxia-inducible factor-1α, and thyroid hormone receptor β1. Those genes are important for cancer cell biological activities (Fig. 1). However, other mechanisms may also involve in thyroxine-integrin αvβ3 signal transduction pathway.

The Wnt/β-catenin pathway is an evolutionarily conserved cell signaling system that mediates key physiological processes but is also incriminated in the occurrence of several malignant neoplasms, including colon cancer. Thyroid hormone has been shown to promote the nuclear accumulation of HMGA2 and β-catenin in a concentration-dependent manner in colorectal cancer cells with different *k-RAS* statuses [61]. A dense collagen matrix increases integrin-mediated cell-ECM interactions with phosphorylated FAK and ERK signaling to exhibit a disrupted membranous E-cadherin/β-catenin complex and, remarkably, show cytoplasmic or nucleic localization of β-catenin, sequentially to regulate cell proliferation in human gastric adenocarcinoma cells [46]. Furthermore, membranous E-cadherin/β-catenin complex could be recovered by inhibiting the phosphorylation of FAK [46]. The nucleus- accumulated β-catenin induces Cyclin D1 and c-Myc [58], the downstream targets of the β-catenin pathway, are also strongly correlated with cell proliferation and cell cycle progression in colorectal cancer[107].

Additionally, T_4_ induces PD-L1 expression in human breast cancer, colorectal cancer, and oral cancer cells [62, 72]. Recently, our studies have shown that thyroid hormone increases cytosolic and nuclear PD-L1 accumulation[10] which may be anti-apoptotic [63]. Expression of *PD-L1* is regulated via activated ERK1/2 and PI3K [45, 62]. Thyroid hormone-induced PD-L1 is involved in CRC proliferation[45]. Blockage of thyroid hormone binding with integrin αvβ3 can inhibit PD-L1 expression and cell proliferation in CRC cells [45]. On the other hand, inhibition of receptor tyrosine kinase (RTK) is able to reduce *PD-L1* expression and CRC proliferation in K-Ras wild type but not K-Ras mutant CRC cells[45]. These studies further demonstrate that thyroid hormone-activated signal via integrin αvβ3 also cross-talks with the EGFR signal to modulate cancer cell proliferation[5].

### Epidermal growth factor receptor (EGFR) signaling in CRC cells

The structural domains of EGFRs include an extracellular ligand-binding component, a transmembrane component, and an intracellular tyrosine kinase feature. The EGFR is activated upon binding with ligands such as EGF, transforming growth factor (TGF)-α, amphiregulin, heparin-binding EGF, and betacellulin [41]. After being bound with ligands, EGFR dimerizes, auto-phosphorylates, and consequently activates the tyrosine kinase component of EGFR [91]. Ultimately, EGFR signaling has positive downstream effects in terms of increased cell proliferation and improved cell survival. The EGFR pathway contributes importantly to cell differentiation, as well as proliferation. A dearth of EGFR activity results in the developmental failure of multiple organs.

Overexpressed EGFRs exist in many primary cancers including CRC, and play important roles in both tumor growth and progression [56]. Expression or upregulation of the EGFR gene was demonstrated in up to 80% of CRC cases [85, 99]. Regular EGFR activity is also crucial for the formation of tumors in adenomatous polyposis coli (APC)-mediated intestinal tumorigenesis [110]. Essentially, EGFRs’ signaling is able to accelerate proliferation, survival, invasion, and immune evasion in CRC cells [12]. Consequently, there is also a metastatic risk [81]. EGFR signaling pathway can regulate migration and invasion through β-catenin activity. Additionally, tumor cells with a low EGFR expression have low tumor metastatic risk and better survival rates in CRC patients [44].

Main EGFR downstream effectors are molecules involved in the Ras-Raf-mitogen-activated protein kinase (MAPK) kinase (MEK)/MAPK pathway. EGF binds to EGFR to promote activation downstream of Ras signaling[118]. Binding to their plasma membrane receptors, growth factors may activate receptor-linked tyrosine kinases (RTKs), leading to activation of Son Of Sevenless (SOS), a Ras-selective guanine nucleotide exchange factor (RasGEF) that supports nucleotide exchange, and an activated conformation of Ras-GTP. When activated, the Ras-GTP complex attaches to a variety of effector proteins involved in downstream signaling and consequent cell growth/survival, differentiation, and both migration and adhesion. Downregulation of the EGFR signaling pathway should, therefore, result in interruption of this pathway and ultimately in reduced cellular proliferation.

Mutations of *K-Ras*, such as G12C, are found in most of pancreatic cancers, and one-third of lung cancers, and 50% of CRCs; these mutations are associated with high mortality rates. Accumulations of abnormal *APC*, *K-Ras*, and *β-catenin* genes are early events in CRC tumorigenesis [15, 60]. However, any correlations that exist among these events are still unclear. EGFR signaling is able to crosstalk with the Wnt-β-catenin pathway to stimulate CRC growth and can trigger β-catenin signals via the receptor tyrosine kinase-PI3K/Akt pathway, while β-catenin can stimulate EGFR signaling via the transmembrane Frizzled receptor [2, 106]. Furthermore, the EGFR signal can crosstalk with β-catenin to promote frequencies of invasiveness and metastasis of cancer cells [2]. EGF-induced nuclear localization of SHC Binding and Spindle Associated 1 (SHCBP1) activates β-catenin signaling by enhancing the CBP/β-catenin interaction [73] and promotes cancer progression [73]. EGFR activation is partly due to α2,6 sialylation of the EGFR by ST6Gal1, which affects EGF-induced cancer cell proliferation [96]. Additionally, ST6Gal1-induced α2,6 sialylation is critical for adhesion and migration of CRC cells [96]. ST6Gal1 induces mutant EGFR sialylation in CRC HCT116 cells [5]. The anticancer activity of gefitinib is more significant in ST6Gal1-deficient CRC cells, as over-expressed ST6Gal1 was shown to suppress gefitinib-induced cytotoxic effects and promote gefitinib-mediated chemoresistance in CRC cells [5].

### Crosstalk between integrin αvβ3 and epidermal growth factor receptor (EGFR) signaling in CRC cells

Thyroid hormone regulates *K-Ras* expression [40]. The hormone significantly enhances expression of *PCNA*, *Cyclin D1,* and *c-Myc* and their protein levels in both *K-Ras* wild type HT-29 and mutant HCT 116 cells [59]. The T_4_ antagonist and derivative of tetrac, nano-diaminotetrac (NDAT), and cetuximab significantly suppress transcription of cell proliferation-associated genes; these include *PCNA*, *Cyclin D1*, *c-Myc,* and *RRM2* induced by thyroxine; these effects are significantly enhanced over cetuximab, alone, in HCT 116 cells. In addition, T_4_ suppression of transcription of mRNAs of pro-apoptotic genes p53 and RRM2B is significantly antagonized by the combination of NDAT and cetuximab compared to cetuximab alone [59]. In *K-Ras* mutant HCT 116 cells, but not in the *K-Ras* wild type COLO 205 cells, the combinations of tetrac/NDAT and cetuximab significantly reduced cell proliferation compared to cetuximab, alone. In summary, T_4_ promotes CRC cell proliferation and this action is opposed by tetrac and NDAT. The combination of tetrac/NDAT and cetuximab potentiates cetuximab actions in K-Ras mutant colorectal cancer cells [59]. These results suggest indicated existence of crosstalk between thyroid hormone and the EGFR-K-Ras signal pathway in CRC.

### Therapies based on targeting EGFR signaling in CRC

EGFR-targeted therapies have been of particular interest because of the clinical benefits conferred by monoclonal antibodies (mAbs) to the receptor, such as panitumumab and cetuximab, and identification of biomarkers that inform treatment decision-making [50]. Genetic heterogeneity in CRC, however, often conveys a need for personalized chemotherapeutic protocols. Genetic variations may make difficult the full characterization of resistance mechanisms in standard therapies [116]. *K-Ras* has been the subject of extensive drug-targeting endeavors over the past three to four decades. These endeavors include targeting the *K-Ras* protein itself, as well as its posttranslational modifications, membrane localization, protein–protein interactions, and downstream signaling pathways. Despite optimized patient selection based on *Ras* mutation status, the primary and secondary resistance to mAbs is still higher than desired [50].

Using molecular targeted drugs, such as bevacizumab, cetuximab, panitumumab, aflibercept, and regorafenib, can increase clinical survival rates [79, 102]. Although new chemotherapeutic regimens have improved patient responses, their use remains limited by inherent chemoresistance of tumors and the acquisition of resistance in the course of therapy [103, 113]. However, anti-EGFR therapies are often affected by tumor cell mutation associated with resistance based on alterations in EGFR-driven signaling systems [113].

Monoclonal antibodies (mAbs) have been extensively investigated for CRC treatment. Cetuximab and panitumumab are mAbs that inhibit activities of EGFR through blocking the binding of EGF to EGFR, including downstream signaling that is initiated at the receptor. Such signaling pathways include Ras-Raf-MEK-MAPK, phosphatase, and tensin homolog (PTEN) and the phosphatidylinositol-AKT pathways [12, 34, 83]. Panitumumab and cetuximab both are in clinical use for CRC [97, 122]. Cetuximab (Erbitux^®^) is a chimeric [immunoglobulin G1 (IgG1)] mAb. When bound to the extracellular domain of the EGFR, cetuximab can block endogenous ligand binding and inhibit proliferation of cancer cells. Cetuximab may also have immune-regulated anticancer effects, for example, antibody-dependent cell-mediated cytotoxicity [83]. In a Phase II clinical trial, cetuximab improved survival and reversed chemoresistance in patients with refractory mCRC [16], a result that led to U.S. Food and Drug Administration (FDA) approval of the drug for management of metastatic CRC. In addition to improving the survival rate, cetuximab maintains the quality of life for mCRC patients [49]. Cetuximab is administered intravenously after initial biweekly or weekly loading dosage and used as a solo agent in the setting of mCRC or in conjunction with a second standard chemotherapeutic agent [78]. A humanized IgG2 EGFR antibody, panitumumab is bound by the EGFR extracellular domain and interrupts signaling for ligand-mediated proliferation. The efficacy of panitumumab was shown to result in clinical benefits both when added to chemotherapy and as monotherapy in mCRC in various clinical settings [1, 29].

The most likely basis for resistance to anti-EGFR therapy in cancer cells is constitutive activation of signaling pathways linked to EGFR and this may or may not be a function of constitutive EGFR activity. The principal predictors of cetuximab failure are point mutations of the KRAS gene, principally in codon 12 or 13 in exon 2 [3, 92]. Functionally, this means that cetuximab monotherapy or conjunctive therapy is to be used in mCRC patients whose tumors bear wild-type (WT) K-ras. After treatment with cetuximab, however, biochemical convergence may occur in tumor cells to reactivate the Ras-Raf-MEK-MAPK signaling pathway [113, 114].

Another EGFR-targeted therapy involves TKIs. TKIs are small molecules derived from quinazolines that can be transported across cell membranes and block the intracellular tyrosine kinase domain of various receptors such as EGFR, Erb2, and vascular endothelial growth factor receptor (VEGFR) [123]. Gefitinib (Iressa^®^) is an EGFR specific antagonist that can block the phosphorylation of the EGFR [47]. It also can target other pathways such as ERK1/2 phosphorylation in mesothelioma cell lines [32]. Gefitinib is used to treat non-small cell lung cancer and various types of cancers as a single agent or in combination with other anticancer agents [7]. It is only used for phase II clinical trial in CRC in Europe [4]. Erlotinib is a specific inhibitor of the EGFR that can also block phosphorylation of the ligand-induced EGFR. Both of these drugs have been highly effective in other tumor types, particularly lung cancer harboring mutations of the EGFR gene [86]. As such, there has been great interest in determining the efficacy of EGFR TKIs in mCRC.

Gefitinib-inhibited EGFR activity results in EGFR dephosphorylation, HER3-phosphatidylinositol 3-kinase (PI3K) complex dissociation, and a decrease in Akt activity [93]. Plasma membrane integrins, ADAM (a disintegrin and metalloproteinase protein), and EGFR have been shown to contribute to fibronectin (FN) induction by the activation of ERK1/2, p38, and Akt. These agents also are involved in promoting growth and invasiveness of cancer cells. Gefitinib prevents FN-induced signal molecule activation and other activities in hepatocellular carcinoma CBO140C12 cells, suggesting that activation of EGFR tyrosine kinase regulates these FN responses [80]. Thus, a gefitinib-induced anti-metastatic activity involves blockage of FN-induced signaling [80]. Gefitinib inhibits activation of Akt and ERK [7] by disturbing the K-Ras/PI3K and K-Ras/Raf complexes to reduce synthesis of matrix metalloproteinases (MMPs). However, constitutive activation of PI3K or ERK1/2 signal transduction pathways is involved in gefitinib-induced resistance in cancers. Gefitinib disrupts K-Ras/PI3K and K-Ras/Raf complexes in human non-small cell lung cancer (NSCLC) Calu3 cells but not in K-Ras-mutant Calu3_ras_ cells [7, 30]. The K-Ras mutation was correlated with gefitinib resistance [95]. Gefitinib combined with lovastatin downregulates the K-Ras protein and can effectively suppress EGFR phosphorylation and activation of Raf, ERK1/2, and Akt in gefitinib-resistant human NSCLC A549 and NCI-H460 cells [7]. EGFR mutations can also affect the sensitivity of CRCs to gefitinib, but this effect is not consistent [125]. Gefitinib was shown to inhibit human chondrosarcoma proliferation and metastasis by inducing cell cycle arrest, leading to a decrease in the migration capacity [109]. Gefitinib also reduces expressions of metastasis-related proteins, such as basic fibroblast growth factor (bFGF) and MMP-2 and MMP-9 [109]. Gefitinib has been combined with other cancer chemotherapeutic agents to manage various cancers [36, 52, 111, 112]. What is clear is that gefitinib affects a number of therapeutic targets in cancer cells mentioned above, yet resistance to this TKI does develop [76]. In this review article, we describe a new treatment strategy that restores responsiveness to gefitinib.

In addition, immunotherapies have been applied in current mCRC studies against other targets. These include use of antibodies that target the VEGF/VEGFR pathway [Bevacizumab (Avastin^®^), and Ramucirumab (Cyramza^®^)]. Alternatively, immunotherapy may use checkpoint PD-1/PD-L1 inhibitors such as Nivolumab (Opdivo^®^) and Pembrolizumab (Keytruda^®^).

### Tetrac derivatives compete with thyroid hormone to bind on integrin αvβ3

Tetrac derivatives compete with T_4_ for the iodothyronine receptor on the integrin αvβ3 [5]. NDAT acts primarily at the cell surface receptor and does not enter the nucleus when internalized by tumor cells. In contrast, tetrac may undergo nuclear uptake and, in the nuclear compartment, tetrac has low-grade thyrometic activity, rather than anti-thyroid (anti-T_4_) effects. Tetrac derivatives block binding of T_4_ to the cell surface thyroid hormone receptor on integrin αvβ3; they thereby inhibit the non-genomic effects of thyroid hormone-initiated downstream signal transduction pathways [5, 59, 64, 72, 90]. The interaction between tetrac derivatives and integrin αvβ3 regulates gene expression related to cancer cell survival pathways, for example, pathways that oppose induction of apoptosis in cancer cells. Tetrac derivatives also downregulate cancer cell proliferation via integrin αvβ3 in the absence of T_4_ [64].

Tetrac and NDAT also support apoptosis and suppress angiogenesis by differentially modulating transcription of a panel of genes linked to these processes[19]. Both tetrac and NDAT upregulate expressions of the proapoptotic BcL-x short form [38], the antiangiogenic thrombospondin 1 (THBS1), and other proapoptotic genes [64]. In addition, they suppress transcription of several anti-apoptotic gene families. Catenin proteins play roles in cell–cell adhesion, and β-catenin also has transcriptional functions in the nucleus. Mutation and overexpression of β-catenin occur in a variety of cancers, including CRC and breast and ovarian cancers [51, 119]. Tetrac and NDAT increase transcription of the *CBY1* gene which codes for an inhibitor of β-catenin [89]. Tetrac and NDAT also reduce β-catenin abundance via downregulation of the *CTNNA1* and *CTNNA2* genes [19]. While the function of CTNNA1 protein may include suppression of invasiveness of tumor cells [115], mutated CTNNA1 may be involved in induction of GI tract cancer [28]. Mutated CTNNA2 is linked to tumor invasion [31]. At the tumor cell surface thyroid hormone analogue receptor on integrin αvβ3, tetrac inhibits the pro-angiogenic activities of vascular endothelial growth factor (VEGF) and basic fibroblast growth factor (bFGF) [18]. NDAT inhibits transcription of anti-apoptotic factors such as myeloid cell leukemia sequence 1 (MCL1) and XIAP. NDAT acts differentially, however, to upregulate expression of apoptosis-inducing genes such as *caspase-2(CASP2)*, *BCL2L14,* and *BAD* [19]. NDAT also blocks transcription of the Ras-oncogene family [19]. The expression of cyclin genes is also downregulated in cancer cells by NDAT [38]. Interestingly, our studies also indicated both tetrac and NDAT are able to inhibit programmed cell death/ligand 1 *PD-L1* expression and protein accumulation by cancer cells [59]. Production of PD-L1 blocks host immune T cells from attacking the tumor cells. The anti-PD-L1 activities of tetrac and NDAT could potentially be a new therapeutic strategy for cancer immunotherapy. NDAT inhibits expression of *ribonucleotide reductase regulatory subunit M2* (*RRM2*) that is caused by the stilbene, resveratrol but potentiates resveratrol’s anticancer activity [90]. In summary, tetrac derivatives regulate expression of genes involved in modulating angiogenesis and regulating tumor cell metabolism by multiple mechanisms [21]. In addition to antiproliferation, tetrac and NDAT were shown to augment other drug-induced anticancer growth [65, 89, 91, 103]. The effects of tetrac derivatives are summarized in Table 2.

**Table 2:** The effects of tetrac derivatives

**Cell cycle** [5, 59, 89]	**Angiogenesis** [5, 18, 38, 89]	**Others** [5, 19, 59, 89, 90]
* CCND1* ↓ (Tetrac, NDAT)	Anti-angiogenic:	* HIF1A* ↓ (tetrac, NDAT)
	* THBS1* ↑ (tetrac, NDAT)	* TP53* ↑ (NDAT)
**Cell proliferation** [5, 19, 38, 59, 89]		* RRM2B* ↑ (NDAT)
* CBY1* ↑ (tetrac, NDAT)	Angiogenic:	* p21* ↑ (NDAT)
* CTNNA1* ↓ (NDAT)	* VEGFA* ↓ (tetrac, NDAT)	
* CTNNA2* ↓ (NDAT)	* bFGF* ↓ (tetrac, NDAT)	
* CTNNB1* ↓ (tetrac)		
β*-catenin* ↓ (tetrac)	**Metastasis** [5, 89]	
* PCNA* ↓ (tetrac, NDAT)	* MMP-2* ↓ (tetrac, NDAT)	
* c-Myc* ↓ (tetrac, NDAT)	* MMP-9* ↓ (tetrac, NDAT)	
*EGFR* ↓ (NDAT)	* MMP-13* ↓ (tetrac)	
**Apoptosis** [5, 38, 59, 66]	**Immune checkpoint** [45]	
Anti-apoptotic:	* PD-L1* ↓ (NDAT)	
* MCL1* ↓ (NDAT)		
* XIAP* ↓ (tetrac, NDAT)	**Chemo sensitization** [5, 89]	
	* HMGA2* ↓ (tetrac)	
Proapoptotic:	* ST6Gal1* ↓ (NDAT)	
* BCL2L14* ↑ (NDAT)		
* CASP2* ↑ (NDAT)		
* BAD* ↑ (NDAT)		

### Combined treatment of tetrac derivatives and anticancer agents

Treatment with tetrac and NDAT is not cytotoxic to nonmalignant cells [19] or in animal studies [5, 90]. We have studied in several cell models the combined treatment effects of tetrac or NDAT as well as other anticancer drugs in CRC cells [5, 59, 68, 89] and other cancer cells [68].

Gefitinib has been shown to be less effective in CRC compared to other cancer types [4]. Compared to results in non-small cell lung cancer (NSCLC) patients, CRC patients required a higher dosage of drug to achieve stale disease, and the latter was not associated with reduction in tumor size [4]. Cellular studies indicated that atorvastatin (5 μM) enhanced cytotoxicity of gefitinib-related inhibition of Akt and ERK activity [7]. Cytotoxicity can be additive in combination therapy.

Functional sialylation of β-galactoside α-2,6-sialyltransferase 1 (ST6Gal1) on the EGFR was highly correlated with CRC progression and metastasis [96]. Upregulation of α2,6-sialylation may also induce radioresistance in CRC [96]. Other studies have shown that gefitinib is more effective in ST6Gal1-knockdown CRC SW480 cells [96]. Our investigation has shown that ST6Gal1 induces sialylation of mutant EGFRs in CRC HCT116 cells [5]. Interestingly, gefitinib increased antiproliferation in ST6Gal1-deficient CRC cells [5]. In contrast, ST6Gal1 overexpression decreased the cytotoxic effect of gefitinib [96]. Sialylation of the EGFR by ST6Gal produced gefitinib chemoresistance in CRC cells [96]. EGFR sialylation affected EGF-mediated cancer cell proliferation [96]. On the other hand, sialylation promoted gefitinib resistance in CRC cells [5]. NDAT reduced ST6Gal1 expression and inhibited CRC cell proliferation [5]. NDAT enhanced gefitinib-induced antiproliferation via a mechanism involving inhibition of ST6Gal1 activity and PI3K activation [5].

Cetuximab (Erbitux^®^) inhibited K-Ras WT cells, but not *K-Ras*-mutant CRC cell growth [59]. Tetrac significantly enhanced cetuximab-reduced cell proliferation in K-Ras-mutant HCT 116 cells, but not in K-Ras WT COLO 205 cells [59]. However, NDAT potentiated cetuximab-induced antiproliferation in both K-Ras WT and *K-Ras*-mutant CRC cells [59]. Gefitinib blocks Akt and ERK activities [7] by disturbing the K-Ras/PI3K and K-Ras/Raf complexes to reduce synthesis of matrix metalloproteinases (MMPs) [112]. Gefitinib (1 μM) did not inhibit PI3K activation in HCT116 cells, although gefitinib inhibited the complexing of K-Ras/PI3K and K-Ras/Raf in NSCLC K-Ras/PTEN or K-Ras/PIK3CA co-mutant cells [7]. Consistent activation of the PI3K/Akt and/or Ras/ERK pathways was associated with gefitinib resistance in NSCLC cell lines [48].

In addition to reducing ST6Gal1 expression, NDAT blocks EGFR sialylation by ST6Gal1 and consequent PI3K activation [5]. When intact—in the absence of NDAT—both reactions contribute to proliferation in *K-Ras* WT and *K-Ras* mutant cells [81]. The combination of NDAT and gefitinib in CRC cell lines permitted efficient identification of pro-apoptotic and metastasis-relevant genes affected by the drugs [81]. NDAT differentially regulates the expression of specific genes at integrin αvβ3 [19, 20, 38, 64] and the consequences of NDAT action are cell cycle disruption, apoptosis, and anti-angiogenesis [20]. Other studies of HCT116 CRC xenograft-bearing mice have also demonstrated that NDAT additively promotes gefitinib-induced anticancer activity [5]. While downregulation of ST6Gal1 transcription has been shown to stimulate tumor cell proliferation both in vitro and in vivo [96], NDAT demonstrated its capability to decrease ST6Gal1 expression and CRC growth. Although decreased ST6Gal1 may increase EGF-induced EGFR phosphorylation and ERK1/2 activation in CRC cells [96], NDAT has been shown to reduce ERK1/2 activation and ST6Gal1 accumulation in CRC cells [5]. In addition, NDAT suppressed PI3K activation to down-regulate *PD-L1* expression and protein accumulation in vitro and in xenograft in *K-Ras*-mutant CRC [45]. Gefitinib effectively reduces cancer metastasis by downregulating expressions of metastasis-linked proteins, e.g., MMP-9, MMP-2, and bFGF [109]. In contrast. NDAT can inhibit expressions of *MMP-2*, *MMP-9*, and *VEGF-A* [19, 64, 66] and further enhance inhibitory effects on *MMP-2*, *MMP-9*, and *VEGF-A* by gefitinib.

**Fig. 2:**
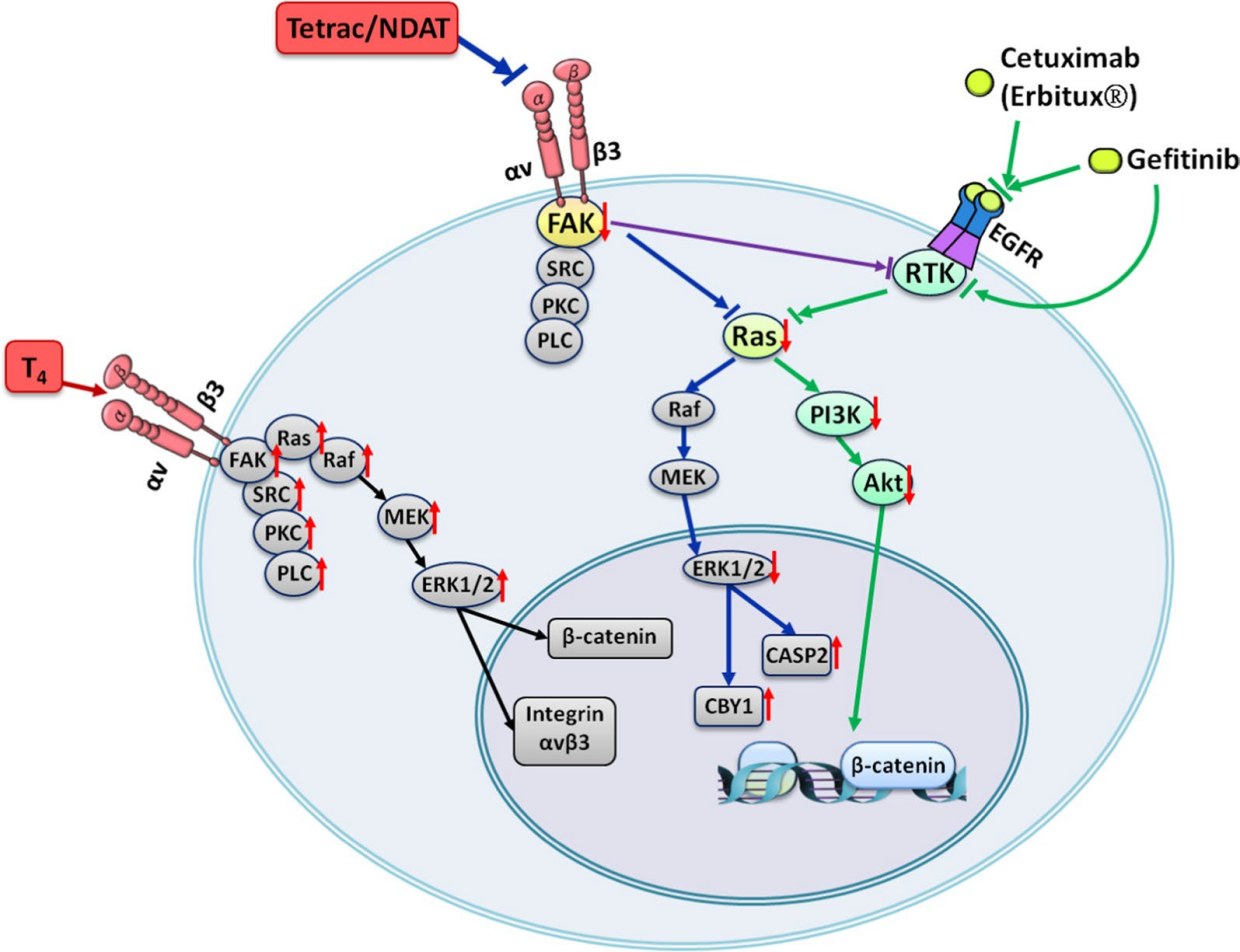
Targeting Therapies of CRC is compensated by NDAT in *K-Ras* Mutant Colorectal Cancers. Thyroid hormone stimulates signal pathway of integrin αvβ3-FAK axis and proliferation. EGF via EGFR-Ras pathway promotes proliferation. It also cross-talks with integrin αvβ3 signal via FAK activation. These signals can induce activation of PI3K- and ERK1/2-dependent pathways. In addition, signals via growth factor receptors are also able to induce β-catenin-dependent cell proliferation. NDAT inhibits signal pathway of integrin αvβ3-FAK axis and proliferation. EGFR-dependent signal pathways via Ras-PI3K/ERK1/2 crosstalk with FAK. These signals can be intercepted by blocking activation of FAK, PI3K and ERK1/2. Crosstalk between growth factor receptors and FAK can be blocked by NDAT treatment

Tetrac derivative actions in cells exhibit potential for the clinical treatment of *K-Ras*-mutant CRC patients. Our studies indicate that NDAT has greater therapeutic potential than tetrac since it can reverse *K-Ras*-mutant-dependent resistance using cetuximab and gefitinib. However, xenograft weights in animals treated via NDAT alone did not significantly decrease compared to those in the untreated control [5, 90]. Therefore, NDAT alone or combined with a low dosage of cetuximab and gefitinib has new chemotherapeutic potential. Such observations show that added or enhanced effects can be obtained when tetrac derivatives are combined with other chemotherapeutic agents (Fig. 2).

## Conclusion

Thyroid hormone as T_4_, acting via cancer cell plasma membrane integrin αvβ3, induces cell proliferation, and metastasis. The hormone may engage in crosstalk with EGFR in modulating a variety of cancer cell activities. Targeting EGFRs by antibodies or by EGFR-specific TKIs has shown promising results in CRC therapies. However, both immunotherapy and targeting therapy in *K-Ras*-mutant CRC patients have raised concerns about resistance. Combined treatment with EGFR-specific inhibitor agents augments antitumor responses beyond initial single EGFR inhibitor therapy [124]. Multiple-agent treatments of cancers have been practiced for years, often achieving efficacy that exceeds single agents. Targeting cell surface integrin αvβ3, tetrac, and chemically-modified tetrac (NDAT) also inhibit the EGFR-dependent signal transduction pathway via crosstalk between the integrin and the EGF receptor. These agents can potentiate the antiproliferative actions of cetuximab and gefitinib in *K-Ras*-mutant CRC. Both gefitinib and NDAT inhibit proliferation in *K-Ras* WT CRC cells. While gefitinib is unable to suppress cell growth in *K-Ras*-mutant CRC cells, NDAT induces anti-proliferation by blocking ST6Gal1 activity and PI3K signal transduction. Although NDAT targets the integrin αvβ3 via crosstalk with EGFR signaling, NDAT enhances anti-proliferation induced by gefitinib in CRC cells. A similar observation was obtained with other EGFR blockers such as cetuximab [59]. Tetrac derivatives can overcome mutations in EGFR signal transduction pathways to potentiate cetuximab-induced antiproliferation in *K-Ras*-mutant CRC. Thus, use of NDAT—either alone or combined with other agents, such as gefitinib and cetuximab is a promising approach to treatment of human *K-Ras*-mutant CRC. A summary of the efficacy in cancer cells of currently available clinical agents and potential advantage of combination treatment with tetrac derivatives are listed in Table 3.

**Table 3:** Actions of modificed tetrac in combination with clinical anti-cancer agents Drug

Clinical agents	Efficacy and deficiency	Tetrac/NDAT combination
Chemotherapy		
Fluoropyrimidine	Despite the improved OS, systemic toxicity and tumor resistance are limitations of this therapy [121]	NA
Oxaliplatin		
Targeted therapy		
1. Monoclonal antibodies		
Anti-VEGF/VEGFR:		
Bevacizumab (Avastin^®^)	Chemo-combination therapy is superior to single agent. PIGF or angiopoietin-2 were upregulated in CRC cases resistant to antiangiogenic therapy [39, 54]	NA
Aflibercept (Eylea^®^ and Zaltrap^®^)		
Regorafenib (Stivarga^®^)		
Ramucirumab (Cyramza^®^)		
Anti- EGFR:		
Cetuximab (Erbitux^®^)	Cetuximab (Erbitux®) inhibited *K-Ras* WT but not *K-Ras*-mutant CRC cell growth[58]	NDAT potentiated cetuximab-induced antiproliferation in both *K-Ras* WT and *K-Ras* mutant CRC cells[58]. They also showed potentiation effect in vivo
Panitumumab (Vectibix^®^)		
Immune checkpoint inhibitor:		
Pembrolizumab (Keytruda^®^)	Pembrolizumab and Nivolumab displayed good efficacy for high levels of microsatellite instability (MSI-H) or MMR deficiency (dMMR) but unsatisfactory results for MS stable and MMR proficient cases. [57, 94]	NA
Nivolumab (Opdivo^®^)		
Ipilimumab (Yervoy^®^)		
2. Small molecules		
EGFR inhibitor:		
Gefitinib (Iressa^®^)	Gefitinib was shown less effective in CRC compared to other cancer types[45]	NDAT enhanced gefitinib-induced antiproliferation via a mechanism involving inhibition of ST6Gal1 activity and PI3K activation[5, 45]
Erlotinib (Tarceva^®^)		

## Data Availability

Not applicable.
